# Supplementary material for: Comparative Efficacy and Safety of Dupilumab and Benralizumab in Patients with Inadequately Controlled Asthma: A Systematic Review
Source: Int J Mol Sci. 2020 Jan 30;21(3):889. doi: 10.3390/ijms21030889 (PMC7037967; doi:10.3390/ijms21030889)
Supplement: Supplementary file 1 [file ijms-21-00889-s001.pdf]

International Journal of
Molecular Sciences

MDPI

Supplementary Information

Supplementary Table S1

Characteristics and key inclusion criteria for the studies included in this analysis. The intension to treat (ITT) population were patients who were randomized regardless of whether an intervention was performed. In LIBERTY ASTHMA QUEST, the placebo had a volume of 2.00 ml which matched the higher dose of dupilumab, and 1.14 ml which matched the lower dose of dupilumab.

Study name	Study duration (weeks)	Treatment arms	Number of patients（ITT）	Key inclusion criteria
LIBERTY ASTHMA QUEST [21]	52	･dupilumab 300 mg (loading dose, 600 mg) s.c. e2w[a]	633	･≥500 μg ICS
		･dupilumab 200 mg (loading dose, 400 mg) s.c. e2w	631	･≥1 asthma exacerbation in the previous 12 months
		･Placebo 2.0 ml [a]	321	･≥12% or ≥$FEV_{1.0}$ reversibility
		･Placebo 1.14 ml	317	･≥1.5 ACQ-5 score[b]
		/total	1902	
CALIMA [24]	56	･benralizumab 30 mg e8w (first three doses e4w) [a]	441	･≥500 μg ICS
		･benralizumab 30 mg every 4 weeks	425	･≥2 asthma exacerbation in the previous 12 months
		･Placebo[a]	440	･≥12% or ≥200 ml of $FEV_{1.0}$ reversibility
		/total	1306	･≥1.5 ACQ-6 score[b]
SIROCCO [23]	48	･benralizumab 30 mg e8w (first three doses e4w)[a]	398	･≥500 μg ICS
		･benralizumab 30 mg every 4 weeks	399	･≥2 asthma exacerbation in the previous 12 months
		･Placebo[a]	407	･≥12% or ≥200 ml of $FEV_{1.0}$ reversibility

/total	1204	▪≥1.5 ACQ-6 score[b]

[a] treatment groups included in this analysis

[b] the 5-item Asthma Control Questionnaire (ACQ-5) or the 6-item Asthma Control Questionnaire (ACQ-6) represents the patient's-reported measure of symptoms of asthma control and change in symptoms due to treatment.

Supplementary Table S2
Search strategies
Source: Pubmed
Searched on: April 20, 2019

#1	Search asthma [MeSH Terms]	122493
#2	Search asthma [Title/Abstract]	140734
#3	#1 OR #2	166708
#4	"dupilumab " [Supplementary Concept]	116
#5	Search (dupilumab)	332
#6	Search (SAR231893)	117
#7	Search (anti–IL-4)	789
#8	Search (anti–interleukin-4)	65
#9	Search (dupixent)	22
#10	#4 OR #5 OR #6 OR #7 OR #8 OR #9	1139
#11	"benralizumab " [Supplementary Concept]	57
#12	Search (benralizumab)	150
#13	Search (MEDI-563)	154
#14	Search (anti–IL-5)	515
#15	Search (anti–interleukin-5)	130
#16	Search (Fasenra)	8
#17	#11 OR #12 OR #13 OR #14 OR #15 OR #16	717
#18	Search antibodies, monoclonal, humanized [MeSH Terms]	47550
#19	#10 OR #17 OR #18	49151
#20	Randomized Controlled trial [Title/Abstract]	61356
#21	Controlled clinical trial [Title/Abstract]	12979
#22	Randomized [Title/Abstract]	476493
#23	Placebo [Title/Abstract]	202573
#24	Randomly [Title/Abstract]	310074
#25	Trial [Title/Abstract]	545083
#26	Drug Therapy [Title/Abstract]	46861
#27	Groups [Title/Abstract]	1931613
#28	#20 OR #21 OR #22 OR #23 OR #24 OR #25 OR #26 OR #27	2735222
#29	#3 AND #19 AND #28 Filters: Humans	348

Results: 348
